# Point-of-care ultrasound in COVID-19 pandemic

**DOI:** 10.1136/postgradmedj-2020-137853

**Published:** 2020-05-13

**Authors:** Sanjeev Bhoi, Ankit Kumar Sahu, Roshan Mathew, Tej Prakash Sinha

**Affiliations:** Emergency Medicine, All India Institute of Medical Sciences, New Delhi, Delhi, India; Emergency Medicine, All India Institute of Medical Sciences, New Delhi, Delhi, India; Emergency Medicine, All India Institute of Medical Sciences, New Delhi, Delhi, India; Emergency Medicine, All India Institute of Medical Sciences, New Delhi, Delhi, India

A multifold increase in patient volume presenting to emergency departments (EDs) during a pandemic demands escalation of surge capacity, including radiological services. In previous pandemics such as severe acute respiratory syndrome and Middle East respiratory syndrome, increased requirement for chest imaging had led to significant overloading of patients in the ED.^1 2^ As point-of-care ultrasound (POCUS) has been demonstrated to identify, in real time, various pathologies of the lung, integration of it was done during patient care in these pandemics and was found to be a great adjunct to clinical decisions.^1 2^

**Figure 1:**
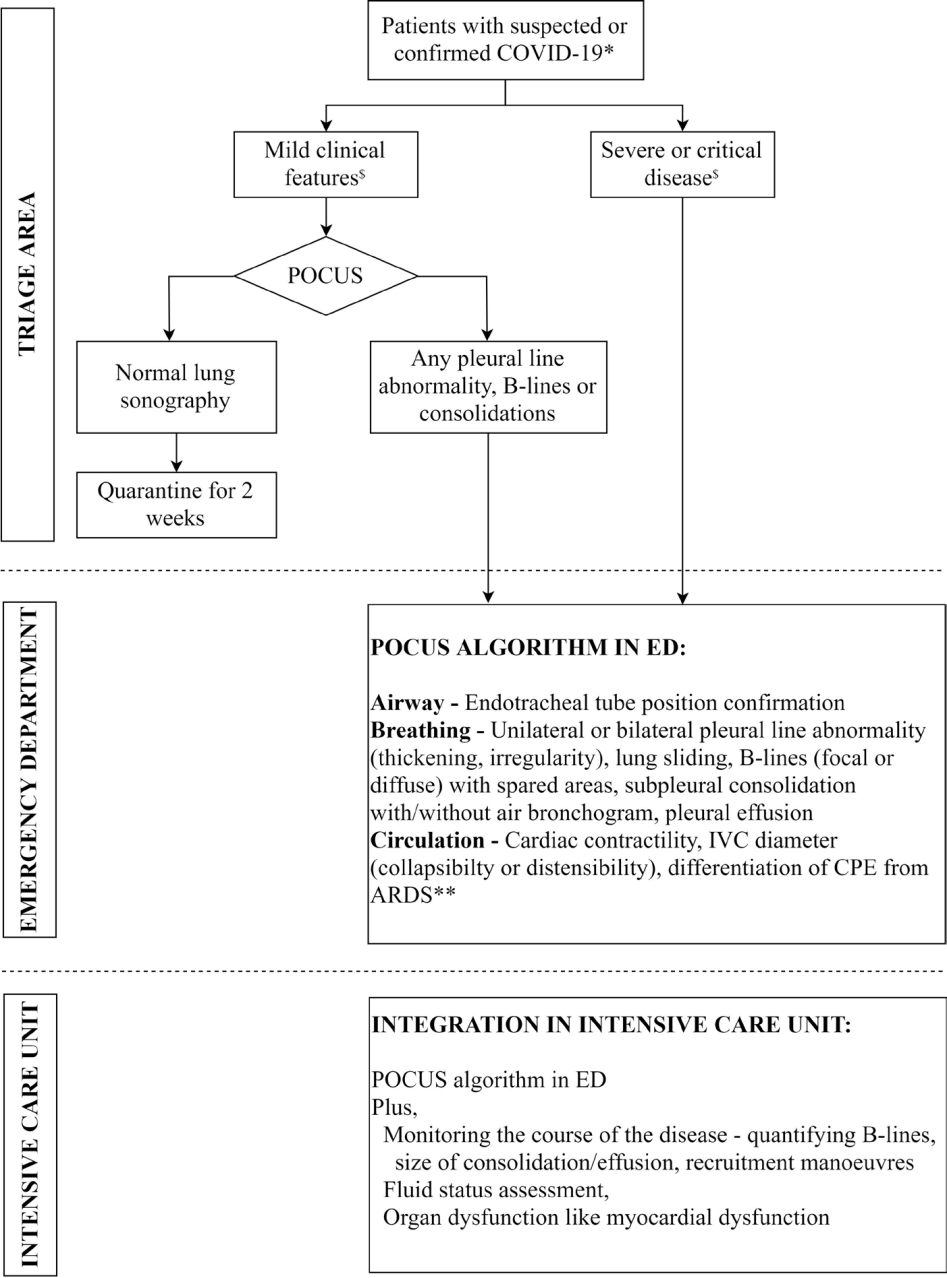
Integration of POCUS in COVID-19 patient management. *Suspected or confirmed COVID-19 case on the basis of recent WHO case definitions. ^$^Severity of cases: mild disease: no pneumonia or pneumonia; severe disease: dyspnoea or tachypnoea (respiratory rate>30 breaths/min); or critical disease: respiratory failure, septic shock or multiorgan dysfunction. **Differentiation of ARDS from CPE on the basis of presence of bilateral pleural line abnormality (thickening>2 mm, coarse pleural line), small subpleural consolidations, ‘spared areas’ in between B-lines, ‘lung pulse’ and reduced or absent lung sliding. ARDS, acute respiratory distress syndrome; CPE, cardiogenic pulmonary oedema; ED, emergency department; IVC, inferior vena cava; POCUS, point-of-care ultrasonography.

Very few case reports and case series have been published regarding the use of POCUS in the recent pandemic of COVID-19. In a case series of 20 patients with confirmed COVID-19 by Peng *et al*, lung sonographic findings were pleural line irregularity and thickening, focal B-lines, bilateral diffuse B-profile with spared areas, subpleural consolidation and, rarely, pleural effusion, which were consistent with CT findings.^3^ Similar findings were reported by Huang *et al* 4 in 20 patients, by Poggiali *et al* 5 in 12 patients and by Buonsenso *et al* 6 in a single patient. Additionally, a Doppler study showed poor blood flow in the COVID-19 consolidation, in contrast to abundant blood flow signal in inflammatory bacterial pneumonia.^4^ In this paper, we suggest a protocol of integration of POCUS in the holistic management of patients with COVID-19 (figure 1), from triage to ED to intensive care unit (ICU).

All suspected or confirmed cases of COVID-19 as per WHO guidelines should be triaged on the basis of clinical stability^7^ and institutional triage protocol. Severe (dyspnoea, tachypnoea and hypoxia) or critically ill patients (respiratory failure, septic shock and multiorgan dysfunction) should be sent to ED without POCUS in the triage area. Patients with mild clinical features should undergo POCUS in the triage area for identifying lung pathologies like pleural line abnormalities, presence of B-lines, consolidation and pleural effusion.^6^ If any of the aforementioned is present, the patient should be shifted to ER for further diagnostic evaluation or otherwise can be sent for home isolation safely.

In the ED, POCUS can be integrated in a fashion similar to that of primary survey. During ‘airway’ management, it can be used as an adjunct for endotracheal tube position confirmation. For ‘breathing’ assessment, detailed lung pathologies like pleural line irregularity or thickening, presence of B-lines, consolidations with or without air bronchograms, blood flow in consolidation, pleural effusion and pneumothorax can be assessed.^3 4^ Even complications like acute respiratory distress syndrome (ARDS) can be diagnosed by lung sonography and differentiated from cardiogenic pulmonary oedema on the basis of presence of bilateral pleural line abnormality (thickening≥2 mm, coarse pleural line), small subpleural consolidations, ‘spared areas’ in between B-lines, ‘lung pulse’, and reduced or absent lung sliding.^8^ It can also be used to assess cardiac contractility and fluid status by inferior vena cava diameter as a part of ‘circulation’ management.

Once patients are admitted in the ICU, apart from using the same POCUS algorithm of ED, it can be used to monitor the course of disease like quantifying B-lines in different sonographic lung regions, size of consolidation or pleural effusion, and development or resolution of any other lung abnormalities. Further, lung recruitment manoeuvres for mechanical ventilation of patients with ARDS can be monitored. Even fluid therapy can be tailored on the basis of POCUS findings.^3^

Recognised limitations of lung ultrasonography (USG) are that findings in COVID-19 are non-specific and it cannot detect lesions that are deep within the lung; that is, the abnormality must extend to the pleural surface to be visible with on USG examination. Although sonographic findings of COVID-19 were predominant in posterior and inferior lung fields, it is feasible to use POCUS in patients with ARDS undergoing prone ventilation. Another limitation of lung USG is that is it an operator-dependent technique and needs close contact with the patient, which may contribute to the COVID transmission to the sonographer.

A separate ultrasound machine and probe should be used for COVID-19 patient imaging to prevent cross infection. In the triage area and ED (during low-risk aerosol generating procedures), it is recommended that probe covers (if available) be used and that the surface of the USG machine that come into contact with either the patient or the clinician be disinfected with low-level disinfectants (LLDs; ethyl or isopropyl alcohol, 70%–90%). During high-risk aerosol-generating procedures like endotracheal intubation in the ED and ICU, the machine and its components should be protected with probe covers and draping material such as translucent bags. These covers should be discarded prior to exiting the patient’s room, taking care to avoid cross contamination. LLDs should be used above the probe covers. Portable handheld USG devices are much easier to decontaminate and should be used if available.^9^

POCUS will help in initial screening and segregation of non-severe patients from severe ones.^6^ The utility of POCUS is more marked in temporary healthcare facilities like isolation wards, where availability of a routine X-ray and CT machines is not possible. The evaluating clinician in the ED or ICU can do lung sonography at bedside for evaluation and monitoring, rather than sending the patient to the X-ray or CT room or using a portable X-ray machine, which needs additional manpower. This will limit the potential exposure to other healthcare workers (HCWs) and people during in-hospital transfer. As recommended by the American College of Radiology, the imaging suite should be environmentally cleaned and decontaminated after every contact with suspected patients, which is obviously more tedious than cleaning a portable ultrasound machine.^10^

We therefore suggest that POCUS be used in COVID-19 patient management, starting from the triage area to ICU care. This will address the safe discharge of patients for home isolation, radiological surge capacity and frequent bedside monitoring, and will limit the biological and radiation exposures to HCWs.
